# Gut microbiota modulation in patients with non-alcoholic fatty liver disease: Effects of current treatments and future strategies

**DOI:** 10.3389/fnut.2023.1110536

**Published:** 2023-02-16

**Authors:** Marta Maestri, Francesco Santopaolo, Maurizio Pompili, Antonio Gasbarrini, Francesca Romana Ponziani

**Affiliations:** ^1^Internal Medicine and Gastroenterology-Hepatology Unit, Fondazione Policlinico Universitario Agostino Gemelli IRCCS, Rome, Italy; ^2^Department of Translational Medicine and Surgery, Università Cattolica del Sacro Cuore, Rome, Italy

**Keywords:** MAFLD, NAFLD, gut microbiota, metabolomics, diabetes, diet, bariatric surgery, probiotics

## Abstract

Non-alcoholic fatty liver disease (NAFLD) is frequently associated with metabolic disorders, being highly prevalent in obese and diabetic patients. Many concomitant factors that promote systemic and liver inflammation are involved in NAFLD pathogenesis, with a growing body of evidence highlighting the key role of the gut microbiota. Indeed, the gut-liver axis has a strong impact in the promotion of NAFLD and in the progression of the wide spectrum of its manifestations, claiming efforts to find effective strategies for gut microbiota modulation. Diet is among the most powerful tools; Western diet negatively affects intestinal permeability and the gut microbiota composition and function, selecting pathobionts, whereas Mediterranean diet fosters health-promoting bacteria, with a favorable impact on lipid and glucose metabolism and liver inflammation. Antibiotics and probiotics have been used to improve NAFLD features, with mixed results. More interestingly, medications used to treat NAFLD-associated comorbidities may also modulate the gut microbiota. Drugs for the treatment of type 2 diabetes mellitus (T2DM), such as metformin, glucagon-like peptide-1 (GLP-1) agonists, and sodium-glucose cotransporter (SGLT) inhibitors, are not only effective in the regulation of glucose homeostasis, but also in the reduction of liver fat content and inflammation, and they are associated with a shift in the gut microbiota composition towards a healthy phenotype. Even bariatric surgery significantly changes the gut microbiota, mostly due to the modification of the gastrointestinal anatomy, with a parallel improvement in histological features of NAFLD. Other options with promising effects in reprogramming the gut-liver axis, such as fecal microbial transplantation (FMT) and next-generation probiotics deserve further investigation for future inclusion in the therapeutic armamentarium of NAFLD.

## Introduction

Non-alcoholic fatty liver disease (NAFLD) is considered the liver mirror of systemic metabolic dysfunction, and represents a condition driven by chronic inflammation (1). NAFLD encompasses a wide spectrum of alterations, ranging from non-alcoholic fatty liver (NAFL) to non-alcoholic steatohepatitis (NASH), with fibrosis at different stages up to cirrhosis (2).

Despite less than 10% of people suffering from NAFLD develop liver-related complications, such as cirrhosis and hepatocellular carcinoma (HCC), the economic burden of the disease is heavy. In fact, NAFLD global prevalence is around 25% in the adult population and 7.6% among children. Owing to its high prevalence, NAFLD is the most rapidly increasing cause of end-stage liver disease, HCC and liver transplantation worldwide, being already in USA and Europe the second leading cause of death (3). In addition, NAFLD is associated with an increased long-term risk of fatal and non-fatal cardiovascular events, and the risk of cardiovascular disease (CVD) is proportional to the stage of fibrosis (4).

Thus, the NAFLD umbrella includes a wide heterogeneity of patients, which cannot be defined simply by the absence of alcohol consumption. The term fatty liver disease associated with metabolic dysfunction (MAFLD) has been therefore introduced to define the disease based on its features, precisely the evidence of liver fat accumulation in addition to at least one among: overweight/obesity, type 2 diabetes mellitus (T2DM) or evidence of metabolic dysregulation (such as high blood pressure, altered lipid panel, impaired fasting glucose, or insulin resistance) (5, 6).

Based on these premises, NAFLD is an increasingly emerging global health problem. Understanding the pathophysiology and molecular mechanisms underlying NAFLD is necessary to highlight new therapeutic targets, considering that there are currently no approved drugs for the treatment of NASH and the standard of care is still based on lifestyle modification (2).

In this review, we focus on the impact of the gut microbiota on the molecular mechanisms underlying NAFLD, understanding how current therapeutic approaches used to treat NAFLD/MAFLD and its associated comorbidities may influence the natural history of the disease through gut microbiota modulation. Finally, we point the view to what may become future therapeutic weapons in NAFLD/MAFLD, acting on the gut microbiota.

## Multiple parallel hits hypothesis and the importance of the gut microbiota in NAFLD/MAFLD pathogenesis

The most valuable hypothesis on the development of NAFLD concerns the presence of several parallel factors that simultaneously generate and maintain inflammation, promoting liver damage with the accumulation of fibrosis. The main protagonists are high-fat diet (HFD), lipotoxicity, gut barrier dysfunction, and dysbiosis (7, 8).

**FIGURE 1:**
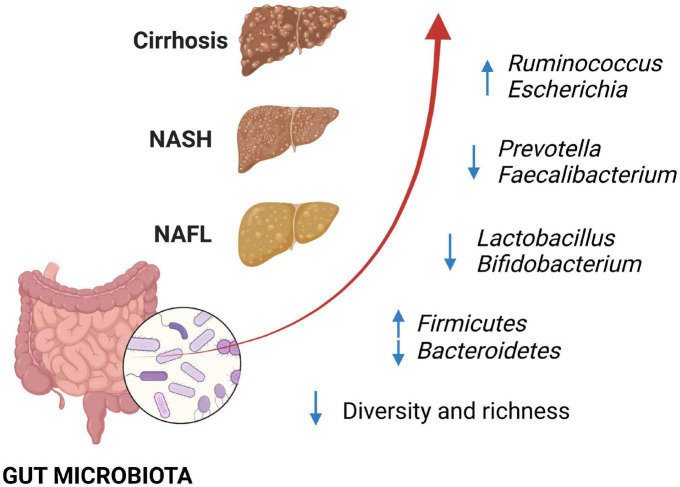
Changes in gut microbiota have been associated with non-alcoholic fatty liver disease (NAFLD), with progressive alteration of some bacterial components, according to the fibrosis degree. Loss of bacterial diversity and depletion of beneficial bacteria such as *Lactobacillus* and *Bifidobacterium* have been described with the progression of NAFLD, together with the increase in *Ruminococcus* and *Escherichia* in patients with advanced fibrosis. NAFLD, non-alcoholic fatty liver; NASH, non-alcoholic steatohepatitis.

In the last decades, the importance of the gut microbiota in the pathophysiology of NAFLD has strongly emerged. Several studies have been carried out to understand the gut microbiota composition. The wide variety of bacteria and the multiple factors which can modify the gut microbiota, included genetic and environmental factors, make the issue hard, with discordant results involving phyla, but also families and genera (9). Of note, some studies correlate the severity of the disease with a specific microbial signature. *Enterobacteriaceae*, including *Escherichia coli* and *Shigella, Bacteroides* and *Ruminococcus* are enriched in patients with moderate-severe fibrosis, while *Faecalibacterium prausnitzii* and *Prevotella* decrease (Figure 1) (10, 11). A recent study by Oh et al. (12) identified some bacteria and bacterial metabolic signatures that independently predict NAFLD-cirrhosis. *Veillonella spp.*, *Enterobacteriaceae* and *Acidaminococcus* correlated positively with the severity of liver fibrosis, whereas *Eubacterium spp.* and *Faecalibacterium prausnitzii* showed opposite trends; in addition, tryptophan, and related metabolites such as indole and kynurenic acid were altered in NAFLD-cirrhosis, with an overall increase in stool tryptophan levels.

Going beyond the mere association between microbial signatures and NAFLD, several studies showed that the gut microbiota is pivotal in inducing the disease phenotype. Indeed, germ-free mice receiving fecal microbial transplantation (FMT) from NASH-affected mice develop hepatic steatosis and inflammation, compared to those receiving FMT from healthy mice (13), on the contrary, FMT from healthy controls protects mice on HFD from intrahepatic lipid accumulation and inflammation (14). Furthermore, FMT from HFD fed mice into pathogen-free mice fed a standard diet, can induce intestinal epithelial barrier (IEB) and gut-vascular-barrier (GVB) derangement (15). This confirms that it is not the type of diet to induce intestinal barrier alteration with consequent bacterial translocation and establishment of inflammatory damage, but rather its influence on the gut microbiota composition. In addition, this study highlighted that the disruption of the intestinal barrier is an early event in the development of NAFLD (15) explaining why liver fat accumulation and lipotoxicity facilitated by insulin resistance (IR) are only components of a wider and more complicated picture. On the other hand, recent studies showed that allogenic FMT from healthy donors can only improve intestinal permeability in patient with NAFLD, with no effect on metabolic parameters (16). Of note, in a mice model of HFD-induced obesity, FMT was able to transmit the beneficial effects of diet and exercise on gut microbiota and metabolic profiles (17).

Noteworthy, gut microbiota exerts a continuous pressure on the immune system, especially when intestinal permeability and bacterial translocation are increased, as it happens in NAFLD (8, 18). Pathogen associated molecular patterns (PAMPs), such as lipopolysaccharide (LPS) from Gram-negative bacteria, bind toll like receptors (TLRs) expressed on epithelial cells and cells belonging to the innate immune system, modulating the inflammatory response against exogenous antigens (19–22). Some preclinical studies have shown a marked involvement of TLR4 and TLR9 in the development of steatosis, inflammation and fibrosis. In fact, TLR4- or TLR9-deficient mice given HFD or choline-deficient diet were protected from hepatic steatosis and inflammation (23, 24). Therefore, dysbiosis associated with many chronic metabolic diseases, producing a continuous immunological stimulation, can promote a condition of low-grade chronic inflammation called meta-inflammation (25, 26).

Therefore, gut microbiota modulation appears crucial in the future treatment of NAFLD/NASH.

## Effect of current NAFLD/MAFLD treatment options on the gut microbiota

### Diet and physical activity

Though several pharmacologic agents have been developed or tested for the treatment of NAFLD, diet still represents the therapeutic cornerstone (2, 27).

**FIGURE 2:**
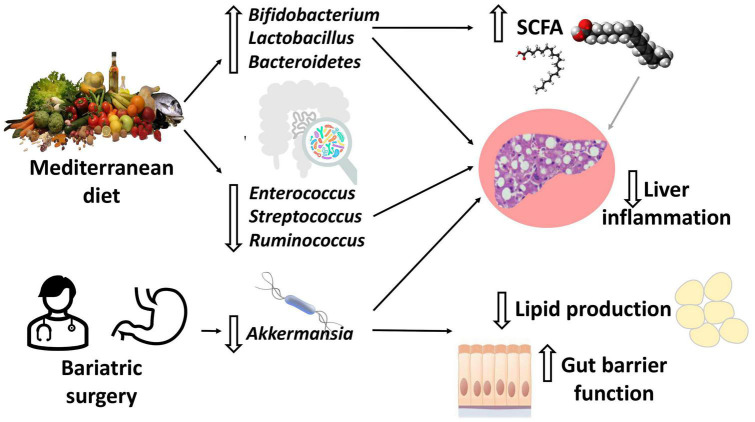
Diet and bariatric surgery effects on the gut microbiota. Adherence to Mediterranean diet (MD) leads to a reduction in intestinal inflammatory bacteria and an increase in beneficial bacteria, including short chain fatty acids (SCFAs)-producing bacteria (mainly *Bifidobacterium* and *Lactobacillus*). Most studies agree on the increase in *Akkermansia* abundance after bariatric surgery (BS). For both MD and BS, the end result is a reduction in inflammation, lipid production and preservation/improvement in the gut barrier function.

It is well-known that the dietary pattern strongly influences the development of NAFLD and other metabolic diseases, but also the gut microbiota (Figure 2). Western diet (WD) rich in refined sugars and saturated fat, mainly based on high red meat consumption and low fish, fruit, vegetables, and fibers intake, has been found to be associated with liver fat deposition (28–32). Conversely Mediterranean diet (MD) based on vegetables and fruit, legumes, aromatic herbs, and extra virgin olive oil as the main source of fat is associated with improvement in metabolic syndrome and intrahepatic fat accumulation (28, 33, 34).

Recently, a clinical trial (35) evaluated the effects of the green-MD, a type of MD further restricted in red and processed meats and particularly enriched in green plants and polyphenols, in a population of patients affected by obesity and dyslipidemia; a significant reduction in liver fat was found with a halving of NAFLD prevalence in the study population. An effect on the gut microbiota was also reported; in particular, at the genus level, eight bacteria were significantly associated with changes in the intrahepatic fat content. Long-term adherence to MD is, in addition, protective against cardio-metabolic diseases, and this effect is greatest among *Prevotella copri* non-carriers in their gut microbiota (36). Fibers, one of the most beneficial and healthy element of MD, improve liver enzymes, lipid panel and fatty liver status in patients with NAFLD, but also improve intestinal permeability (37) and have a great impact on the gut microbiota composition. Overall, fibers intake increases *Bifidobacterium* and *Bacteroidetes*, and decreases inflammatory bacteria such as *Enterococcus, Streptococcus*, and *Ruminococcus* (38). A specific subtype of fibers is represented by resistant starch, which are found in foods such as banana, potatoes and corn, and enhance the growth of SCFAs producing bacteria, such as *Lactobacillus* and *Bifidobacterium* among others (39). SCFAs interact directly with the G protein-coupled receptors (GPR) 41 and 43, also known as free fatty acid receptor (FFAR) 3 and 2, respectively. The main SCFAs agonists of these receptors are acetate, butyrate and propionate, with different affinity (40). Through this interaction, SCFAs trigger anti-inflammatory pathways and the peroxisome proliferator-activated receptor (PPAR)-γ/adenosine monophosphate-activated protein kinase (AMPK) signaling pathway, the latter causing the inhibition of triglycerides and cholesterol production, the increased release of pro-peptide YY (PYY) and glucagon-like peptide 1 (GLP-1), the regulation of appetite and the improvement of intestinal barrier function (39–41). In a preclinical study, inulin fiber administration improved hepatic steatosis and fibrosis in mice, through hepatic free fatty acid receptor 2 (FFAR2)/G-protein-coupled receptor 43 (GPR43) signaling; it should be noted that this effect was mediated by the growth of the SCFAs-producing strains *Bacteroides acidifaciens* and *Blautia producta*, resulting in increased acetate content in the intestinal lumen (42). In a randomized, double-blind, cross-over study, colonic infusion of SCFAs mixtures increased fat oxidation, energy expenditure and PYY levels, also decreasing lipolysis in overweight and obese men (43). Another randomized, controlled, cross-over study demonstrated that ingestion of 10 g inulin-propionate ester significantly increased postprandial plasma PYY and GLP-1 secretion and reduced energy intake over a 24-week period; furthermore, hepatic lipid content and visceral fat deposition were reduced, while weight gain and insulin-resistance were prevented (44). In murine models, administration of sodium butyrate, reduced inflammation and liver steatosis, protecting against WD-induced NASH (45).

Fructose is another crucial dietary element involved in the onset of NAFLD, both by damaging the intestinal barrier and inducing dysbiosis (46–53). Indeed, rats fed with high-sugar-diet show an overall decrease in gut microbiota alpha diversity, as well as a reduction in *Bifidobacterium*, *Lactobacillus*, and members of *Clostridiaceae* family, and an increase in *Coprococcus*, *Ruminococcus*, *Clostridium*, and *Firmicutes/Bacteroidetes* ratio (51, 53, 54).

Meat, yolk, and dairy products are rich in choline, an essential nutrient involved in triglyceride metabolism and necessary for the packaging of very low-density lipoprotein (VLDL) and its export from hepatocytes (55–57). For this reason, a choline deficient diet has been adopted for decades to study NAFLD and NASH in rats (58). As the gut microbiota uses choline for the production of trimethylamine (TMA) (59), contributes to reduce choline bioavailability, mimicking the effects of a choline deficient diet (60). In addition, TMA is oxidized by hepatic flavin monooxygenases to trimethylamine oxide (TMAO) before being released into circulation (61). Noteworthy, higher levels of TMAO were found in NAFLD patients with respect to healthy controls, and correlated with fibrosis stage (62). TMAO reduces cholesterol conversion into bile acids (BAs) altering lipid homeostasis (63), promotes inflammation in adipose tissue and leads to insulin resistance (64), thus favoring the development of NAFLD and NASH. Choline deficiency has been recently linked not only to NASH development, but also to gut microbiota dysbiosis in mice; decreased abundance of *Alistipes*, *Ruminococcaceae, Bifidobacterium, Lactobacillus*, and *Akkermansia*, and increased abundance of *Bacteroides* and *Ruminococcus* were found (65, 66). As mentioned above, *Bifidobacterium* and *Akkermansia* usually have beneficial effects through SCFAs production and modulation of inflammatory response, while *Ruminococcus* is associated with fibrosis in patients with NASH.

Besides diet, physical activity has been proven to be effective in NAFLD treatment, even without weight loss or any dietary change (67–69). Eight weeks of individualized exercise reduce transaminases, markers of inflammation, and improve surrogate scores of steatosis and fibrosis. Furthermore, exercise modifies the gut microbiota, increasing the abundance of *Bacteroidetes* and *Euryarchaeota*, decreasing *Actinobacteria*, and improving richness (70). Both moderate-continuous and sprint-interval training reduce systemic and intestinal inflammation, and improve the gut microbiota profile by reducing *Firmicutes/Bacteroidetes* ratio, and decreasing *Clostridium* and *Blautia* abundance (71). Responders to exercise exhibit gut microbiota enhanced capacity for SCFAs biosynthesis and catabolism of branched-chain amino acids (72). In a 1-year lifestyle intervention with energy-restricted MD plus physical activity and behavioral support, a decrease in several members of *Firmicutes* and a selective increase in SCFAs producers was observed, which was paralleled by weight loss and improved CVD risk (73).

### Bariatric surgery

Bariatric surgery (BS) is the most effective treatment for long-term weight control in obese people (74, 75), and to effectively improve obesity-related comorbidities (74). Epidemiological data report that almost all obese patients and about 75% of overweight people are affected by NAFLD (76). Several studies and meta-analyses demonstrated a significant improvement or even resolution of NAFLD histological features, liver enzymes, glucose tolerance, and lipid panel after BS (76, 77). This beneficial effect results not only from the metabolic consequences of weight loss and visceral adipose tissue reduction, but also from the inhibition of pro-inflammatory cytokines release from adipose tissue, and the reduced supply of free fatty acids (FFAs) to the liver, with a consequent modulation of lipids and glucose metabolism (41). Roux-en-Y Gastric Bypass (RYGB), inducing anatomical changes with hepato-biliary diversion, lowers the concentration of BAs delivered to the colon; while some studies reported either an increase or a reduction in both primary and secondary BAs (78–82), overall it seems that the ratio of primary to secondary BAs decreases regardless of weight loss (83). Furthermore, BS is associated with changes in the release of gastrointestinal hormones, such as GLP-1, gastric inhibitory polypeptide, leptin, PYY, and ghrelin, which are implied in the reduction of appetite and increase in energy expenditure (84).

Changes in the gut microbiota and its related metabolites have been observed after BS, potentially being crucial in NAFLD/NASH improvement or resolution (Figure 2). Published data agree on increased microbial richness after BS, mostly Roux-en-Y Gastric Bypass (RYGB) and Sleeve Gastrectomy (SG) (9, 72, 85–87). There is also broad consensus on the increased abundance of *Akkermansia muciniphila* after both SG and RYGB, and, generally, after weight loss (76, 87–89). *Akkermansia* has been demonstrated to prevent the development of fatty liver disease in mice, reducing the expression of interleukin (IL)-6 and sterol regulatory element-binding protein (SREBP), which is involved in triglycerides synthesis in the liver (90). *Akkermansia* is also a mucin degrader, with the ability to reinforce epithelial barrier, and has been linked with reduction in fat deposition, and protection against insulin resistance and obesity in humans (91). While after RYGB abundance of *Proteobacteria* seems always to increase (78, 88), data about *Firmicutes* and *Bacteroidetes* after both RYGB and SG are discordant (76, 88, 89). A recent meta-analysis investigating human studies and animal experiments involving six different BS techniques, found a reduction in *Firmicutes* abundance with concomitant increase in *Bacteroidetes*, *Proteobacteria*, *Verrucomicrobia*, and *Fusobacteria* (92). Also during the long-term follow-up of nine severe obese patients who underwent biliopancreatic diversion, gastric bypass, or SG, *Enterobacteriaceae* enrichment was observed, while *Clostridiaceae* and *Lachnospiraceae* decreased (86). Interestingly, Tremaroli et al. (93) showed that changes in the gut microbiome did not depend on body mass index (BMI) variation.

However, it is not clear whether changes in the gut microbiota are a mere consequence of the anatomical, hormonal, and metabolic changes, or take part to these modifications contributing to the beneficial effects of BS. The most important proof of concept that the gut microbiota is a main actor in this context is provided by FMT models. Indeed, FMT from mice treated with RYGB to non-operated germ-free mice or from obese patients treated with RYBG or vertical banded gastroplasty to germ-free mice resulted in weight loss and reduced fat mass (93, 94).

### Anti-diabetic drugs

**FIGURE 3:**
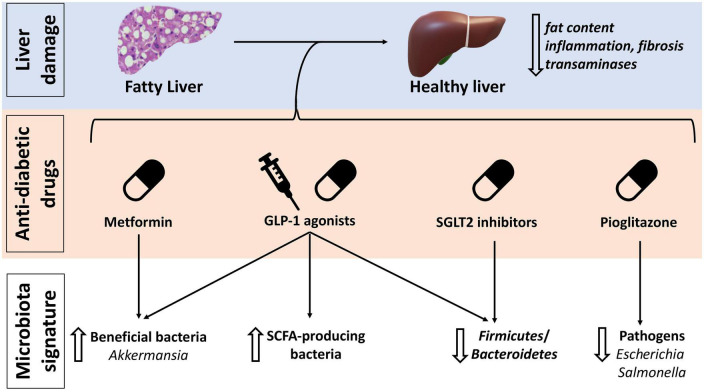
Anti-diabetic drugs effects on non-alcoholic fatty liver disease (NAFLD) and gut microbiota. GLP-1, glucagon-like peptide 1; SCFA, short-chain fatty acids; SGLT, sodium-glucose cotransporter inhibitors.

Due to the strong association between NAFLD and diabetes (6, 95) in absence of drugs specifically approved for the treatment of NAFLD the effect of anti-diabetes drugs on this disease have been object of great interest. Intriguingly, the effect on gut microbiota composition, which is a crucial element in NAFLD pathogenesis and progression, may contribute to their benefit in this setting (Figure 3 and Table 1).

**TABLE 1:** Studies investigating the effect of anti-diabetic drugs and farnesoid X receptor (FXR) modulators on the gut microbiota.

Drug name	Drug class	Study design	Effects on microbiota
Rosiglitazone (102)	PPAR-γ agonist	1 week rosiglitazone administration to mice fed with HFD for 30 days	Restoration of spatial distribution of ileal microbiota
Canagliflozin (111)	SGLT2 inhibitor, SGLT1 inhibitor (modest)	2 week canagliflozin administration to renal failure mice	↑*Bacteroidetes* ↓*Firmicutes* ↓*Actinobacteria*/*Bifidobacterium* ↑ cecal SCFAs content
SGL5213 (112)	SGLT1 inhibitor	2 week SGL5213 administration in normal and adenine-induced renal failure mice	↑*Bacteroidetes* ↓*Firmicutes* ↓*Allobaculum*
Metformin (105)	Hepatic glucose output suppression through AMPK-dependent and independent pathways	4 months metformin administration in a randomized, placebo-controlled, double-blind study in individuals with newly diagnosed T2DM on a calorie-restricted diet	↑ SCFAs producing bacteria (*Bifidobacterium*, *Blautia, Proteobacteria Shewanella)*. ↑ *Akkermansia muciniphila*
TUDCA (146)	FXR modulator	4 week TUDCA administration to mice with HFD-induced NAFLD	↑*Bacteroidetes* ↓*Firmicutes* *↑Faecalibacterium*/*Akkermansia* ↓ *Mucispirillum*/*Ruminococcus gnavus*
Aldafermin (149)	FGF19 analog	12 week aldafermin treatment in patients with NASH in a prospective, phase 2 study	↑*Veillonella* ↓hydrophobic bile acids

PPAR, peroxisome proliferator activated receptors; HFD, high fat diet; SGLT, sodium−glucose co-transporter; SCFA, short chain fatty acid; AMPK, adenosine monophosphate-activated protein kinase; T2DM, type 2 diabetes; TUDCA, tauroursodeoxycholic acid; FGF19, fibroblast growth factor 19; NASH, non-alcoholic steatohepatitis.

Pioglitazone appears to be especially effective in reducing liver fat content, fibrosis and liver enzymes, despite causing an increase in BMI (96, 97). Pioglitazone belongs to PPARs agonists. PPARs are a group of receptors involved in glucose and lipid metabolism and in the anti-inflammatory response in NAFLD/NASH (98–100). They also take part in gut microbiota modulation, being involved in commensal bacteria homeostasis and prevention of the growth of pathobionts such as *Escherichia* and *Salmonella* (99–101). As demonstrated in HFD fed mice, rosiglitazone restores a healthy gut microbiota and improves intestinal permeability after only 1 week of treatment (102).

Metformin has shown some improvement in glucose tolerance, liver function and steatosis, in patients with NAFLD associated or not whit diabetes (96, 103, 104). Its effects are mediated, at least partially, by gut microbiota modulation, as metformin is able to select SCFAs producing bacteria such as *Bifidobacterium*, *Blautia*, and *Shewanella*, increasing also the abundance of *Akkermansia muciniphila* (105).

GLP-1 agonists are another relatively new class of drugs effective in diabetes and also able to ameliorate BMI, liver enzymes and liver fat content in NAFLD/NASH patients (96, 97, 106–109). A recent phase 2 trial on Semaglutide reported 40% of NASH resolution in patients with or without type 2 diabetes (107). Remarkably, in a pre-clinical study, a GLP-1/GLP-2 receptor dual agonist improved BMI, glucose homeostasis, liver triglycerides, liver fibrosis, and intestinal barrier permeability in NASH murine models. In addition, the abundance of SCFAs producing bacteria, in particular *Bifidobacterium*, increased, together with that of several bacteria associated with a healthy phenotype such as *Prevotella*, *Lactobacillus*, and *Akkermansia*; on the contrary *Firmicutes*, implied in obesity, were decreased (Figure 3) (110).

Lastly, sodium-glucose cotransporter 2 (SGLT2) inhibitors have also shown promising results in patients with NAFLD, liver fibrosis and steatosis (98). Canagliflozin, a SGLT2 inhibitor with also a modest inhibitory effect on SGLT1, was found to tendentially increase *Bacteroidetes* and decrease *Firmicutes* abundance, and to increase cecal SCFAs content in mice (111). Selective inhibition of SGLT1 has demonstrated to restore gut dysbiosis in renal failure mice (112, 113).

## Future treatment options for NAFLD/MAFLD: Efficacy comes through the gut microbiota modulation

### Antibiotics

Systemic antibiotics have been one of the first experimented pharmacologic treatment for NAFLD, with beneficial effects on insulin resistance and liver fat accumulation in humans and mice models (48, 114). More recently, broad spectrum antibiotic therapy with metronidazole 1,000 mg per day plus ciprofloxacin 500 mg daily for 1 week, has been proven to reduce plasma levels of TMAO in healthy participants exposed to phosphatidylcholine challenge (115). This supports a possible use of antibiotics as disease modifiers by correction of NAFLD-associated dysbiosis *via* the TMAO metabolic pathway.

Rifaximin, a poorly absorbed antibiotic with eubiotic properties (116), also showed beneficial effects in patients with NAFLD. Some clinical trials in biopsy-proven NAFLD and NASH patients treated with rifaximin 1,100/1,200 mg daily have shown a significant reduction in endotoxin and liver enzymes serum levels, while reduction in BMI was only mild and no changes in the lipid profile were observed (117, 118). In addition, a NAFLD-liver fat score improvement occurred (117).

Rifaximin is currently approved for the treatment of hepatic encephalopathy, and its use is supported by data showing an overall improvement in intestinal permeability, bacterial translocation, and endotoxemia (119, 120). Preclinical studies have investigated the possible mechanisms. Rifaximin directly upregulates the expression of tight junction proteins, mainly zonula occludin-1 (ZO-1), thus lowering intestinal permeability (121, 122). A recent clinical study also showed that rifaximin improves hepatic encephalopathy by suppressing the enrichment of living from the oral cavity mucin-degrading bacteria in the colonic microbiota (i.e., *Veillonella, Streptococcus, Akkermansia*, and *Hungatella*) (120). Among these bacteria, *Akkermansia* is usually known for its beneficial effects including the control of host mucus turnover, layer thickness, and gut barrier preservation (123). However, it is possible that excessive mucus-degrading activity exerted by multiple elements of the gut microbiota can be potentially harmful, and may damage the intestinal barrier.

In mice model of NASH, Jian et al. (124) found that rifaximin modulates gut microbiota and reduces ileal deoxycholic acid, whereas Enomoto et al. (125) found that combination of rifaximin and the pro-kinetic lubiprostone ameliorated intestinal permeability *via* restoring gut epithelial tight junction proteins and counteracting LPS-induced intestinal barrier dysfunction; in addition, the abundance of *Bacteroides*, *Lactobacillus*, and *Faecalibacterium* increased while that of *Veillonella* decreased, resulting in higher levels of SCFAs.

### Next generation probiotics

In the last few decades, several clinical trials investigated the effects of probiotics in NAFLD. Despite good expectations, a recent meta-analysis by Tang et al. (126) showed poor efficacy in reducing body weight, and minor results on the degree of liver fat infiltration, liver enzymes, lipid panel, glucose homeostasis, and pro-inflammatory cytokines. Another meta-analysis reported the superiority of probiotics over placebo in patients with NAFLD on the improvement of BMI, liver tests, and hyperglycemia; however, probiotics failed to ameliorate liver fibrosis and did not seem as beneficial as previously suggested on lipid profile, lacking strong evidence to support a positive effect on inflammation (127). In both meta-analyses *Lactobacillus spp.* and *Bifidobacterium spp.* were the predominant strains investigated. Apart from the modest results, there was a large heterogeneity among studies, with the heavy limitation of the absence of standardization of the currently available probiotic supplements, and no guidance on the best formulations or duration of treatment to adopt, which makes it difficult to interpret the data. A further confusing element are technical limitations; in fact, commercially available probiotics and those tested in previous studies basically have an aerobic metabolism, whereas most beneficial probiotics are anaerobic (128, 129). For these reasons, numerous conflicting data on probiotics can be found in literature.

Nevertheless, research in this field is still ongoing. Starting from the evidence of a lower abundance of *Akkermansia muciniphila* in overweight/obesity untreated type 2 diabetes mellitus or hypertension (130), a randomized, double-blind, placebo-controlled pilot study of daily oral supplementation of 10^10^ *A. muciniphila* bacteria, either live or pasteurized, for 3 months in overweight and obese insulin-resistant subjects was performed (131). No safety issue was reported; metabolic parameters such as insulin sensitivity, plasma total cholesterol, insulin, and BMI improved; in addition, pasteurized *A. muciniphila* led to the reduction of liver enzymes and lipopolysaccharides (LPS) plasma levels, suggesting a strengthening effect on the intestinal barrier. However, no significant change on the overall gut microbiota community was observed, except for the enrichment of *A. muciniphila* abundance. Further studies are needed to bring this new probiotic in clinical practice.

### Fecal microbial transplantation

FMT aims to restore intestinal homeostasis through the administration of a healthy gut microbiota. It can be performed with different techniques, mainly by endoscopic infusion or oral capsules and it is currently approved for the treatment of *C. difficile* infection, although potential application fields are multiple, including liver disorders (132–134).

Benefits of FMT in mice models of HFD-induced steatohepatitis has been demonstrated at many levels. A reduction in liver fat content and intrahepatic pro-inflammatory cytokines, together with improvement in NAS score has been reported after FMT; higher abundance of beneficial bacteria such as *Christensenellaceae* and *Lactobacillus*, increased butyrate cecal content and higher expression of ZO-1 were also documented, along with reduced endotoxemia (14). Histological amelioration of necro-inflammatory features, pro-inflammatory cytokines, and lipid metabolism were found after repeated FMT in humans as well (Table 2) (135). Only 6 weeks after FMT from lean donors to male recipients with metabolic syndrome, a significant increase in gut microbiota diversity and in butyrate-producing intestinal bacteria, as well as an improvement in peripheral insulin resistance was observed (136).

**TABLE 2:** Human studies evaluating the efficacy of fecal microbial transplantation (FMT) or next generation probiotics for the treatment of NAFLD.

Study design	FMT procedure	Follow-up	Results
FMT from healthy lean donors to male subjects with metabolic syndrome (136)	One duodenal infusion	Week 6	↑insulin sensitivity ↑gut microbiota diversity ↑butyrate-producing bacteria
FMT from healthy lean donors with or without LSI, to obese subjects with T2DM (137)	Repeated duodenal infusion every 4 weeks for up to week 12	Week 24	↑ *Prevotella copri* ↑butyrate-producing bacteria ↓*Clostridium clostridioforme*/*Fusobacterium ulcerans* (FMT with and without LSI) ↑*Bifidobacterium spp./Lactobacillus* ↓total LDL cholesterol ↓liver stiffness (FMT with LSI)
Allogenic or autologous FMT from healthy lean donors to patients with NAFLD (16)	One duodenal infusion	Week 6	↓intestinal permeability in patients with elevated small intestinal permeability at baseline (allogenic FMT arm)
FMT from healthy donors to NAFLD patients (138)	One infusion *via* colonoscopy, followed by three enemas over 3 days	Week 4	↓fat attenuation degree ↑gut microbiota diversity ↑*Bacteroidetes* ↓*Firmicutes* ↓*Proteobacteria* Treatment effect of FMT on lean NAFLD was better than that on obese NAFLD
*Akkermansia muciniphila* administration to overweight/obese insulin resistant subjects	Daily oral administration of 10^10^ bacteria for three months, both alive and pasteurized	Month 3	↑insulin sensitivity ↓total cholesterol ↓BMI ↓GGT/AST ↓LPS

LSI, lifestyle intervention; NAFLD, non-alcoholic fatty liver disease; LDL, low density lipoproteins; BMI, body mass index; GGT, gamma-glutamyl transferase; AST, aspartate aminotransferase; LPS, lipopolysaccharide.

A recent randomized, double-blind, placebo-controlled trial including obese subjects with type 2 diabetes demonstrated that FMT *via* oesophago-gastro-duodenoscopy repeated every 4 weeks for up to 12 weeks was safe and enhanced lean gut microbiota engraftment (88.2%) in this population, although better results (100%) were obtained by FMT plus lifestyle intervention (LSI). Notably, only FMT plus LSI led to lipid panel and liver stiffness improvement at week 24 (137).

Another double-blinded randomized controlled trial, conducted in patients with NAFLD, evaluated the effects of autologous or allogenic FMT from healthy donors on insulin resistance, hepatic fat content, and intestinal permeability. After 6 months, no significant benefits were observed, except for an improvement in small intestinal permeability (16). Of note, a recent randomized controlled trial conducted in patients with NAFLD, demonstrated FMT superiority over probiotics in improving liver fat content and gut microbiota composition; a superior clinical efficacy of FMT in lean NAFLD than in obese NAFLD patients was observed (138).

Given the key role of the gut barrier and gut microbiota in the pathogenesis of NAFLD, ongoing studies (Table 3) are trying to better understand the reason for the failures of FMT in this setting and to maximize its efficacy in NALFD patients.

**TABLE 3:** Ongoing trials investigating the application of fecal microbial transplantation (FMT) in non-alcoholic fatty liver disease (NAFLD)/non-alcoholic steatohepatitis (NASH) treatment.

Study	Design	Status	ClinicalTrials.gov identifier
The effect of consecutive FMT on NAFLD	Double-blinded randomized controlled trial, randomization 1:1 to allogenic and autologous gut microbiome transplantation Phase 4	Unknown	NCT04465032
Transplantation of microbes of fecal origin for prevention and treatment of metabolic syndrome and non-alcoholic fatty liver disease	Randomized Phase 1/2	Completed	NCT02496390
Efficacy, safety of intestinal microbiota transplantation for non-alcoholic fatty liver disease	Open label, parallel study	Unknown	NCT03648086
Effects of fecal microbiota transplantation on weight in obese patients with non-alcoholic fatty liver disease	Randomized controlled trial	Recruiting	NCT04594954
FMT in NASH. A pilot study	Single Group Assignment Phase 1	Unknown	NCT02469272
Fecal microbiota transplantation for the treatment of non-alcoholic steatohepatitis, a pilot study.	Single Group Assignment Phase 1	Not yet recruiting	NCT03803540

### Farnesoid X receptor modulation

According to several preclinical studies, BAs metabolism is involved in NAFLD pathogenesis and progression through different mechanisms, including modulation of the farnesoid X receptor (FXR) signaling (139). FXR, with the downstream expression of fibroblast growth factor 19 (FGF19) in human intestine, is implicated in the negative feedback of BAs synthesis (139, 140). Beyond their toxic effect on the liver, BAs modulate the activation of Takeda G protein-coupled receptor 5 (TGR5) which regulates inflammation and glucose homeostasis, by inducing the release of GLP-1. FXR is also involved in glucose and lipid homeostasis, so that its activation promotes glucose uptake, inhibits lipogenesis, and favors fatty acids oxidation (139).

A progressive decrease in FXR expression was found in the liver of healthy controls, patients with NAFLD and patients with NASH, respectively (141, 142); notably, NAFLD-associated dysbiosis is characterized by the overabundance of bacteria producing secondary BAs, which inhibit FXR signaling (8). Indeed, BAs pool presents some differences between NAFLD/NASH and controls. Serum concentration of both primary and secondary BA is increased in NAFLD (143) as well as in NASH, both in fasting and postprandial conditions (144). A four-fold increase in glycocholate and taurocholate, and a two-fold increase in glycochenodeoxycholate were found in NASH, with a tendency for these two BAs to increase also in simple steatosis (145). Remarkably, these differences are associated with changes in the gut microbiota composition. Patients with NASH have a higher abundance of taurine and glycine metabolizing bacteria, compared with healthy patients; moreover, the FXR antagonist DCA was increased, while the agonist CDCA was decreased in NAFLD, explaining, at least partially, the altered FXR-signaling mechanism (143). In mice models of HFD-induced liver steatosis, supplementation with tauroursodeoxycholic acid (TUDCA) counteracts intestinal inflammation and intestinal barrier disruption by increasing the expression of tight junction molecules, antimicrobial peptides, lysozymes, and mucopolysaccharide, reducing serum inflammatory cytokines and intestinal lipid absorption (146). Of note, HFD fed mice showed a gut microbiota composition similar to that associated with obesity and NAFLD, with increased *Firmicutes* and decreased *Bacteroidetes*, whereas HFD fed mice treated with TUDCA presented a gut microbiota composition similar to that of normal diet fed mice, with inverted proportion of *Firmicutes* and *Bacteroidetes* and reduction in *Proteobacteria* (146). In addition, anti-inflammatory taxa such as *Faecalibacterium* and *Akkermansia* were increased, and pro-inflammatory taxa such as *Mucispirillum* and *Ruminococcus gnavus* were reduced in TUDCA treated mice (146), proving the strict connection between BAs, microbiota and liver inflammation.

Positive evidences in mice have led to test FXR modulating agents for the treatment of NAFLD/NASH. In the multicenter, randomized, placebo-controlled phase 3 trial REGENERATE, the FXR agonist obeticholic acid, has been shown to improve the histological features of NASH. According to other previous studies, pruritus and increased LDL cholesterol were the most commonly reported adverse events (147). Another FXR agonist, the non-bile acid agonist MET409, in a 12-week, randomized, placebo-controlled study, ameliorated liver fat content in NASH patients, showing a better and more tolerable profile then others FXR agonists (148). A recent phase 2 study evaluated the effect of aldafermin, an analogue of the intestinal hormone FGF19, on the gut microbiota in patients with NASH. Aldafermin increased the abundance of *Veillonella*, a commensal with lactate-degrading properties, in a dose-dependent manner, and this inversely correlated with serum levels of toxic, hydrophobic BAs. As both the richness and the diversity of the gut microbiota were substantially stable during aldafermin treatment, and only *Veillonella* abundance changed significantly, *Veillonella* has been proposed as a biomarker of treatment response (149).

## Discussion

NAFLD is a worldwide high-prevalent disease, nowadays one of the main causes of chronic liver disease. It is strongly associated with metabolic disorders, and heavily influenced by the gut microbiota for both onset and progression.

A healthy gut microbiota seems to have features that are lost in NAFLD. Indeed, bacterial diversity and richness are reduced, and the proportion of *Firmicutes* increases over *Bacteroidetes*; bacteria able to produce SCFAs from fermentation of dietary fibers, such as *Lactobacillus* and *Bifidobacterium*, are depleted as well as those with anti-inflammatory properties, such as *Faecalibacterium* and *Akkermansia.* Conversely, pro-inflammatory pathobionts such as *Ruminococcus*, *Streptococcus*, *Enterococcus, Shigella*, *Escherichia*, and *Clostridium* are well-represented. The imbalance in BAs pool, in particular the overall increase in BAs and the relative prevalence of secondary BAs, is strictly associated with gut microbiota composition and involved in the alteration of FXR signaling, with detrimental metabolic and toxic effects. Intestinal barrier impairment is another hallmark of NAFLD, being documented since the early stages of the disease.

Several NAFLD features are similar to that induced by WD regimens, while MD is associated with a healthy gut microbiota. Although weight loss and MD are still the cornerstones of NAFLD treatment, other promising treatment opportunities are landing in the NAFLD scenario, and base their efficacy on gut microbiota modulation. BS can lead even to a complete resolution of histological features of NAFLD/NASH; it is associated with an increase in gut microbiota richness and in the abundance of *Akkermansia*, which has anti-inflammatory and positive metabolic properties. However, little is known about the effects of increasing other bacterial phyla, such as *Proteobacteria*, so further studies are necessary to understand the clinical significance. Furthermore, diabetes therapeutic armamentarium is part of NAFLD management, as NAFLD and diabetes share a common metabolic background, and are strictly connected. Diabetes drugs not only ameliorate glucose homeostasis, but also influence the gut microbiota, promoting the growth of healthy bacteria. Other agents such as next-generation probiotics, mainly *A. muciniphila*, or FMT could be valid additional or alternative options to restore a healthy microbiota and modify the course of the disease. Finally, intestinal microbes may also be used as biomarkers of treatment response, such as *Veillonella* during aldafermin treatment.

The evidence discussed in this paper stems from the effort to find a common thread between gut microbiota, NAFLD, and possible therapeutic implications. However, as a final remark, it should be noted that the scientific literature is overflowing with conflicting data. This is because the gut microbiota is extremely complex in its organization, and equally variable from person to person, being strongly influenced by different pathological conditions and multiple environmental as well as socio-cultural factors. Furthermore, the taxonomic identification of bacteria reported by different studies is strictly dependent on the methodology and bioinformatics pipeline used (Supplementary Table 1). Therefore, results derived from similar studies but using different analysis methodologies may produce non-uniform data. Another element of difficulty in bringing order to this vast landscape is the fact that bacteria are often analyzed at high levels in the taxonomic scale, while at the genus and species level they may have opposite behaviors. Each bacterium is also capable of exerting different functions even though it belongs to the same genus or species, making it extremely complex to understand what metabolic results may derive from apparently taxonomically similar bacterial populations. Last but not least, given the high complexity of the microbiota-host-external factors interaction, the end result is made even more fluid and variable. As a function of this, the concepts of “healthy gut microbiota” and “disease signature” are being questioned, as it is not possible to make generalizations that can fit everyone. However, certain ecological features (e.g., a reduced alpha diversity) and specific microbial elements (such as increase of *Enterobacteriaceae* abundance in inflammatory conditions) are recurrent in many studies, and suggest that there is a common denominator or “core” features of the gut microbiota associated with human disease.

## Author contributions

MM reviewed and interpreted literature data and wrote the manuscript. FS and FP interpreted literature data and wrote and revised the manuscript. AG and MP revised the manuscript. All authors read and agreed to the published version of the manuscript.
