# Supplementary material for: Gut microbiota modulation in patients with non-alcoholic fatty liver disease: Effects of current treatments and future strategies
Source: Front Nutr. 2023 Feb 16;10:1110536. doi: 10.3389/fnut.2023.1110536 (PMC9978194; doi:10.3389/fnut.2023.1110536)
Supplement: Supplementary file 1 [file Table_1.DOC]

Supplementary Material

Gut microbiota modulation in patients with nonalcoholic fatty liver disease: effects of current treatments and future strategies

**Marta Maestri^1^, Francesco Santopaolo^1^, Maurizio Pompili^1,2^, Antonio Gasbarrini^1,2^, Francesca Romana Ponziani^1,2*^**

^1^Internal Medicine and Gastroenterology-Hepatology Unit, Fondazione Policlinico Universitario Agostino Gemelli IRCCS, 00168 Rome, Italy.

^2^Translational Medicine and Surgery Department, Università Cattolica del Sacro Cuore, 00168 Rome, Italy.

*** Correspondence:**Francesca Romana Ponziani
francesca.ponziani@gmail.com

# Supplementary Data

**Supplementary Table 1:** sequencing methods used in the various trials to study the microbiota

| DNA SEQUENCING METHODS | 16S rRNA | SHOTGUN |
| --- | --- | --- |
|  | Shen F et al, Hepatobiliary Pancreat Dis Int 2017 (11)  Crescenzo R et al, Food Nutr Res 2017 (53)  Sen T et al, Physiol Behav 2017 (54)  Ye JZ et al, World J Gastroenterol 2018 (65)  Schneider KM et al, Int J Mol Sci 2019 (66)  Motiani KK et al, Med Sci Sports Exerc 2020 (71)  Muralidharan J et al, Am J Clin Nutr 2021 (73)  Ilhan ZE et al, NPJ Biofilms Microbiomes 2020 (78)  Juárez-Fernández M et al, Nutrients 2021 (86)  Dao MC e al, Gut 2016 (91)  Liou AP et al, Sci Transl Med2013 (94)  Byndloss MX et al, Science 2017 (101)  Tomas J et al, Proc Natl Acad Sci U S A 2016 (102)  Kim ER et al, Hepatology 2022 (110)  Mishima E et al, Am J Physiol Renal Physiol 2018 (111)  Depommier C et al, Nat Med 2019(131)  Zhou Det al, Sci Rep 2018 (14)  Xue L et al, Front Cell Infect Microbiol 2022 (138)  Wang W et al, Br J Pharmacol 2018 (146)  Loomba R et al, Hepatology 2021 (149) | Loomba et al, Cell Metab 2017 (10)  Oh et al, Cell Metab 2020 (12)  Wang DD et al, Nat Med 2021 (36)  Tremaroli V et al, Cell Metab 2015 (93)  Wu H et al, Nat Med 2017 (105) |
